# Smartphones as new tools in the management and understanding of Parkinson’s disease

**DOI:** 10.1038/npjparkd.2016.6

**Published:** 2016-03-03

**Authors:** Andrew D Trister, E Ray Dorsey, Stephen H Friend

**Affiliations:** 1 Sage Bionetworks, Seattle, WA, USA; 2 Center for Human Experimental Therapeutics, University of Rochester Medical Center, Rochester, NY, USA

“Variability is the law of life...”—Osler, 1903.^1^

Parkinson’s disease (PD) has been traditionally characterized by the motor symptoms of disease such as tremor, altered gait, and bradykinesia, with a well-established pathophysiology related to loss of dopaminergic neurons in substantia nigra. Nevertheless, the large variability in presentation and progression of disease, and a constellation of additional non-motor symptoms related to the disease make diagnosis and management of PD dependent on expert clinical assessment and patient report. In the absence of a quantifiable laboratory or imaging biomarker, the goal of precision medicine as applied to PD will be impossible to achieve. Despite the efforts of engineers and physicians over the past 90 years to build mechanical and electronic devices to assess the severity (and change) of symptoms such as tremor, these objective measurements fail to capture the complexity of disease as they collect a single time-point measurement of a single sign. In this chronic and progressive illness, assessing function (such as with activities of daily living) is also of great importance, and the efforts to include these in common rating scales such as the UPDRS provide a brief episodic view into the lives of patients. Consequently, the complexity of the disease, and the variability between and within patients, cannot be captured with these simple evaluations.

We propose that a novel approach using sensors on increasingly pervasive technology, such as smartphones, can make ecologically valid assessments of function for patients with PD. Such a system would ideally evaluate all aspects of disease through a series of measures made on activities and through passive inference. The combination of rich streams of nearly continuous data from individuals can provide a more detailed view into the day-to-day variability that patients describe. Performing such assessments in the ‘real world’ without the control of a laboratory setting has the potential to accentuate ‘noise’ in any measurements; however, the inclusion of many data points over time can reveal subtle trends before those change might be clinically evident. Ultimately, such an approach necessitates sophisticated analytical pipelines to provide quality assurance and measures before these could be used for clinical decision-making. We term the outcome of these analyses ‘clinically actionable phenotypes’ mirroring the ‘clinically actionable mutations’ that have been the focus of decades of research in oncology.

The first steps toward collecting these ecologically valid clinically actionable phenotypes have been taken in PD. On the basis of work pioneered by the Kinetics Foundation,^2,3^ Max Little and colleagues conducted a feasibility study to measure five different motor dimensions among PD patients using an Android smartphone app.^4^ On the basis of their positive experience classifying participants in the pilot study, we embarked on developing an iOS-based study leveraging Apple’s ResearchKit.^5^ The resultant study and app, *mPower*,^6^ leverage a novel asynchronous and remote consent process to enroll participants, and within weeks, over 15,000 participants enrolled in *mPower*. In addition to surveying participants with a subset of questions from the Movement Disorder Society-Universal Parkinson's Disease Rating Scale (MDS-UPDRS), participants were asked to perform short activities, such as speeded tapping for 20 s on the screen of the phone or phonating a vowel for 10 s into the microphone, multiple times a day. Sensors within the phone are used to ‘record’ each of these activities. For those participants with PD and taking medication, the timing of each recording with relation to their last dose of medication is also evaluated. By virtue of evaluating performance using sensors, a quantitative assessment can be made along many different dimensions. For example, in addition to reporting the total number of taps on the screen performed in 20 s (a reasonable surrogate for bradykinesia), the accuracy of each tap related to targets on the screen can also be assessed (a potential surrogate for tremor).^7^

In a preliminary analysis for the first 6 months of data from those who used the app most, predictions of the effect of medication from these features reveal distinct patterns phenotypes. These individualized approaches to classifying patients and the impact that treatments have on their symptoms give the first glimpse of the power of these approaches. To take these initial findings and ensure they are robust and actionable in the clinic, we believe that a larger group of researchers should have access to coded study data to make new insights. One of the key tenets in the design of these mobile health studies is that the data that are generated belong to the participants, and they have the choice of whether there data are shared in this way. An overwhelming majority of participants in *mPower* have said that their data can be used for future research, and we have made the data available for other research^8^ and through https://www.synapse.org/mPower.

The path forward to the endstate of clinically actionable phenotypes will require large collaborative efforts. Rather than working in traditional academic or industry silos, where ideas are shared only after they are proven successful, we believe that a community of patients, their families, neurologists, advocates, and researchers should iterate and work together to define the ways that new technologies can be most powerful for PD. All of the code for this study is open and freely licensed,^6^ and we continue to build new features to capture a richer view into the lives of people with PD. We hope that this community will continue to grow and flourish to explore the impact that such approaches can have on PD and adjacent movement disorders.
